# How did the use of psychotropic drugs change during the Great Recession in Portugal? A follow-up to the National Mental Health Survey

**DOI:** 10.1186/s12888-020-02620-1

**Published:** 2020-05-11

**Authors:** Manuela Silva, Ana Antunes, Sofia Azeredo-Lopes, Graça Cardoso, Miguel Xavier, Benedetto Saraceno, José Miguel Caldas-de-Almeida

**Affiliations:** 1 Comprehensive Health Research Centre (CHRC), Lisbon Institute of Global Mental Health. Nova Medical School, Nova University of Lisbon. Rua do Instituto Bacteriológico, n°5, 1150-190, Lisbon, Portugal; 2 Nova Medical School, Nova University of Lisbon. Campo Mártires da Pátria, 130, 1169-056, Lisbon, Portugal; 3 Chronic Diseases Research Centre (CEDOC). Nova Medical School, Nova University of Lisbon. Rua do Instituto Bacteriológico, n°5, 1150-190, Lisbon, Portugal

**Keywords:** Psychotropic drugs, Economic recession, Gender, Age, Public health

## Abstract

**Background:**

Research suggests that economic recessions might be associated with a higher use of psychotropic drugs, but literature is scarce and contradictory in identifying the most vulnerable groups. This study aims to assess possible changes in the use of psychotropic drugs due to the economic recession in Portugal, by comparing self-reported consumption in 2008/09 and 2015/16.

**Methods:**

Data from the World Mental Health Survey Initiative Portugal (2008/09) and the National Mental Health Survey Follow-Up (2015/16) were used (*n* = 911). McNemar’s tests were performed to estimate changes in consumption of any psychotropic drug and of antidepressants, anxiolytics, and hypnotics/sedatives. Multiple Generalised Estimating Equations models with interaction effects were used to estimate the population odds of consuming psychotropic drugs according to year, gender and age.

**Results:**

An increase of 6.74% was estimated in the consumption of psychotropic drugs from 2008/09 to 2015/16. Population odds of consuming any psychotropic drugs in 2015/16 were estimated to be 1.5 times higher than in 2008/09 (OR = 1.50;95%CI:1.13–2.01), particularly for hypnotics/sedatives (OR = 1.60;95%CI:1.14–2.25). Women and older individuals presented higher odds of consuming any psychotropic drugs (OR = 2.79;95%CI:2.03–3.84, and OR = 1.80;95%CI:1.28–2.54), after adjusting for year of assessment and education. However, when evaluating the interaction effect of the year with gender and age, men and younger individuals reported higher odds of consuming any psychotropic drugs in 2015/16, when compared to 2008/09 (OR = 1.85;95%CI:1.08–3.17, and OR = 1.95;95%CI:1.32–2.90, respectively).

**Conclusions:**

The findings indicate that the period of economic recession was associated with an increased risk of psychotropic drugs use in Portugal. Consumption of psychotropic drugs remained higher among women and older individuals, but the results suggest that the economic crisis had a disproportionate impact on men and younger individuals. This identification of the most vulnerable population groups is useful to design effective and targeted public health interventions aimed at alleviating the effects of economic recessions.

## Background

The 2008 global financial crisis precipitated the most severe economic recession to date, surpassing the Great Depression of the 1930s [1, 2]. Among European countries, Portugal was particularly affected, in terms of decline in gross domestic product (GDP), rise of unemployment rates, and government deficit [3, 4]. As part of the austerity policies, large cuts to public expenditure and to health and social budgets were made, and savings of €670 million were demanded from the Portuguese National Health Service, targeting care and drug expenditure, prescriptions, workforce, and user charges [3]. A mix of cost-containment policies in the pharmaceutical sector was implemented, aiming to reduce the public expenditure on drugs from 1.55% of GDP in 2010 to 1% by the end of 2013 [3, 5, 6]. Measures included increase in co-payments for pharmaceuticals, generic drugs promotion campaigns, electronic prescription, and discounts granted to the public payer [3, 6, 7]. These measures tend to shift the cost-burden to those who needed medicines, in a country where out-of-pocket payments already represented an important part of total health care expenditure [8, 9]. Concerns arose about the unintended risk of less equitable access to needed medicines and “cost-related non-adherence” [10, 11].

The impact of economic crises on the use of mental health care is expected to be mixed. On the one hand, demand for mental health is likely to increase, and substantial research has shown that periods of economic recession can be damaging to mental health due to risk factors such as economic adversity (e.g. job and income loss) [12–15]. On the other hand, mental health systems may not meet this growing need, due to fiscal austerity measures that reduce availability and affordability of services [12]. Most findings suggest that during recessions prescriptions for psychotropic drugs rise [16–19], including those to treat depressive and anxiety disorders [18, 20–23]. Some studies didn’t find this association [24, 25], but found a widening of consumption differences according to gender and age [25].

Several studies have examined patterns and trends of consumption of psychotropic drugs over the past decades, showing overall increases in utilization, consistently higher among women and with older age, lower income and educational levels, and mental health care use within the past 12-months [26]. Some possible explanations for gender differences in the use of psychotropic drugs were pointed out, such as representing a proxy for differences in the prevalence of mental disorders in women and men, reflecting the degree of gender inequality within a country, or denoting different healthcare-seeking behaviour, prescription preferences by mental health professionals and services, or health expenditure allocated to mental health care [26]. Among classes of psychotropic drugs, antidepressants are the most widely and increasingly prescribed drugs, particularly among women and across older age groups [27].

Little is known about the impact of the Great Recession on changes in the pattern of consumption of psychotropic drugs in Portugal. Compared to other European countries, Portugal has higher rates of consumption of psychotropic drugs [28, 29], which may be partly explained by the fact that the country has one of the highest prevalences of mental disorders in Europe [30, 31]. This high consumption has been recognized as a public health challenge [28], as it is largely based on anxiolytics, and hypnotics/sedatives. Available official data show a continuous increase in the prescription and dispensing of all subgroups of psychotropic drugs in the National Health Service between 2000 and 2016, especially antidepressants and antipsychotics [28, 32]. This may reflect a greater accessibility to medicines, longer use, approval of new therapeutic indications [28], and the deterioration of the population’s mental health, particularly common mental disorders. The increase in the prescription and utilization of psychotropic drugs since the beginning of the economic recession suggests that the worsening of mental health problems and the need for medication exceeded the impact of changes in affordability. Available research evaluating the impact of pharmaceutical sector policies during this period focuses exclusively on consumption of antipsychotic drugs [7].

Given the scarce evidence and the public health importance of this topic in the Portuguese context, this study aims to assess possible changes in the use of psychotropic drugs indicated for the treatment of common mental disorders, the clinical situations predictably most affected by economic recessions [13, 14]. Self-reported consumption of psychotropic drugs, including antidepressants, anxiolytics, and hypnotics/sedatives, was evaluated before and after the economic recession in Portugal, accounting for gender and age differences. This research adds to the existing literature by comparing the use of psychotropic drugs by the same individuals before and after an economic recession, and the findings may provide valuable insights for targeted interventions and policy-making.

## Methods

### Design and study sample

This study used data from the National Mental Health Survey (T0) and the National Mental Health Survey Follow-Up (T1).

#### National Mental Health Survey (T0)

The National Mental Health Survey was conducted in 2008/09 as part of the World Mental Health Survey (WMHS) Initiative. This nationally representative cross-sectional survey was based on a stratified multistage clustered area probability household sample of Portuguese-speaking adults, aged 18 years or above, residing in permanent dwellings in the country’s mainland.

A response rate of 57.3% was obtained, similar to the results in Belgium, France, Germany, and the Netherlands. The survey was administered by trained lay interviewers with a computer-assisted personal interview in a face-to-face setting, and the questionnaire was divided into two parts to reduce respondent burden. Part I was administered to all participants (*n* = 3849), and Part II to participants with criteria for any mental disorder, and to a probability sample of 25% randomly selected participants who did not meet these criteria (*n* = 2060). Part I included core diagnostic assessment of mental disorders, and Part II included the assessment of additional mental disorders, correlates and consequences of mental disorders, self-reported chronic conditions, and use of services.

Two different weightings were considered. Weighting procedures were applied to Part I data to adjust differential probabilities of selection between and within households, non-response bias and discrepancies between the sample and the sociodemographic and geographic distribution of the Portuguese census population. Part II data were additionally weighted to adjust for differential sampling of Part I participants into Part II [33].

Informed consent was obtained from all respondents and all procedures were approved by the Ethics Committee of the Nova Medical School, Nova University of Lisbon (ref.: 10/2008). Further details regarding the study design, fieldwork procedures, and methodology can be found elsewhere [33].

#### National Mental Health Survey Follow-up (T1)

In 2015/16, a follow-up of the National Mental Health Survey was conducted to compare epidemiological data on mental disorders, socioeconomic conditions, and use of services before and after the economic recession. Informed consent was obtained from participants and all procedures were approved by the Ethics Committee of the Nova Medical School, Nova University of Lisbon (ref.: 16/2015/CEFCM).

Fieldwork procedures were similar to those of the WMHS. All individuals with a mental disorder diagnosis in T0 and a 20% random sample of those without a diagnosis that had participated in Part II were recruited to the follow-up survey (*n* = 911). A new weighting was created based on the Part II weighting previously described, to adjust for the differential probability of selection to the follow-up [34].

### Measurements

#### Assessment of psychotropic drugs

The use of any psychotropic drugs in the previous 12 months, regardless of the presence of a clinical diagnosis, was evaluated in both T0 and T1. In both T0 and T1, participants were asked the same question: “Did you take any type of prescription medicine in the past 12 months for problems with your emotions, substance use, energy, concentration, sleep, or ability to cope with stress? Include medicines even if you took them only once”. If so, participants were requested to indicate which of the medicines they had taken from a long list that included 1) antidepressants, 2) anxiolytics, and 3) hypnotics/sedatives.

#### Sociodemographic characteristics

Participants’ sociodemographic characteristics, including gender, age, and educational level, were evaluated at baseline (T0). Age was assessed as a continuous variable and dichotomized into two categories (18–49 years of age versus > 50 years of age at the baseline). Education is widely used as an indicator of socioeconomic position in epidemiological studies [35], and the number of years of educational attainment at the baseline (continuous variable) was used to adjust multivariate models.

### Statistical analysis

Frequency tests and McNemar’s tests for comparing marginal proportions were used for descriptive analyses. Multiple Generalised Estimating Equations (GEE) models were performed to estimate the population odds of consuming psychotropic drugs according to year, gender and age groups. The correlation between the observations among the paired measurements were considered as having an exchangeable structure, meaning that the correlations are identical but unknown [36].

The choice of the GEE models in this study was made since the same individuals were considered in T0 and T1 (i.e. repeated measures) and because of the interest in evaluating changes at the population level.

Odds ratios (OR) were estimated and interpreted at specific levels of the main effects and interaction terms considering differences in psychotropic drugs in both periods according to gender and age. The standard errors of the odds ratio estimates, used to obtain the confidence intervals, employed values from the variance-covariance matrix of the corresponding model fits. Estimates were weighted according to the characteristics of the study, as previously explained. A significance level of α = 0.05 was used throughout the analysis. Data analysis was conducted using R version 3.5.1. The R package geepack was used to fit the GEE models [37, 38].

## Results

The characteristics of the study sample at the baseline are presented in Table 1.

**Table 1:** Characteristics of the study sample

	**N (%)**^a^
Gender	
Men	328 (49.6)
Women	583 (50.4)
Age	
18–49 at baseline	545 (59.8)
≥ 50 at baseline	366 (40.2)
	**Mean (sd)**^a^
Education (years)	9.22 (4.83)

^a^%, N unweighted; means and standard deviations (sd) estimated with weighting from follow-up study

The results of the McNemar’s tests, presented in Table 2, indicate a significant increase in the percentage of individuals consuming any psychotropic drugs between T0 and T1 (6.74; 95%CI: 3.89–9.6). Statistically significant increases in the consumption of any psychotropic drugs were found among men (7.97%; 95%CI: 4.23–11.71), women (5.54%; 95%CI: 1.25–9.84), and younger individuals (9.85%; 95%CI: 5.9–13.79). Regarding specific types of psychotropic drugs, an estimated increase of 2.80% in the percentage of individuals reporting consumption of antidepressants was found from T0 to T1 (95%CI: 0.65–4.95). A statistically significant increase was also found for women, estimated around 3.75% (95%CI: 0.18–7.33). No statistically significant increase in the consumption of antidepressants was found among men, but the confidence interval obtained is marginally close to zero on its lower margin, which may suggest a tendency for an increase among this group. A statistically significant increase between T0 and T1 was also found in the percentage of younger individuals consuming antidepressants, estimated at around 4.72% (95%CI: 1.81–7.62). The percentage of individuals reporting consumption of hypnotics/sedatives had a statistically significant increase from T0 to T1, estimated at around 4.81% (95%CI: 2.30–7.31). The percentage of males reporting use of hypnotics/sedatives was also estimated to have increased around 7.30% (95%CI: 3.96–10.62). This tendency was also found in the percentage of younger individuals (18–49 years at baseline), estimated at around 5.84% (95%CI: 2.72–8.95). A statistically significant increase between T0 and T1 was found in the percentage of younger individuals (18–49 years at baseline) taking anxiolytics, estimated at around 4.73% (95%CI: 1.21–8.24).

**Table 2:** Estimates of the use of psychotropic drugs in 2009, 2015 and the difference between those years

	**Use in 2009 (%)**	**Use in 2015 (%)**	**Difference between 2009 and 2015 and respective 95%CI (%)**^**a**^
**Any psychotropic drug**			
**Population**	20.9	28.2	6.74 (3.89–9.60)^b^
**Gender**			
Men	11.5	19.1	7.97 (4.23–11.71)^b^
Women	30.9	37.2	5.54 (1.25–9.84)^b^
**Age**			
18–49 at baseline	15.3	25.7	9.85 (5.90–13.79)^b^
≥ 50 at baseline	29.8	32.0	2.21(−1.76–6.18)
**Antidepressants**			
**Population**	8.3	11.0	2.80 (0.65–4.95)^b^
**Gender**			
Men	3.6	5.4	1.81 (−0.53–4.17)
Women	12.9	16.6	3.75 (0.18–7.33)^b^
**Age**			
18–49 at baseline	7.9	12.5	4.72 (1.81–7.62)^b^
≥ 50 at baseline	9.1	8.9	0.28 (−3.40–2.84)
**Anxiolytics**			
**Population**	12.3	14.5	2.36 (−0.32–5.04)
**Gender**			
Men	7.8	11.4	3.41 (−0.05–6.87)
Women	16.9	17.7	1.33 (−2.75–5.42)
**Age**			
18–49 at baseline	8.9	13.4	4.73 (1.21–8.24)^b^
≥ 50 at baseline	17.4	16.3	1.11 (−5.24–3.03)
**Hypnotics/sedatives**			
**Population**	11.4	16.9	4.81 (2.30–7.31)^b^
**Gender**			
Men	5.4	12.6	7.30 (3.96–10.62) ^b^
Women	17.6	21.1	2.21(−1.51–5.92)
**Age**			
18–49 at baseline	7.0	13.8	5.84 (2.72–8.95)^b^
≥ 50 at baseline	18.2	21.5	3.31 (−0.86–7.47)

^a^ McNemar’s test

% weighted

^b^Statistical significance considered when 95%CI does not contain 0

*CI* Confidence interval

The results of the GEE models, presented in Table 3, indicate that the population odds of consuming any psychotropic drugs in T1 were estimated to be 1.5 times higher compared to T0 (OR = 1.50; 95%CI: 1.13–2.01), after adjusting for age, gender and education. Likewise, the population odds of consuming hypnotics/sedatives in T1 were estimated to be 1.6 times higher than in T0 (OR = 1.60; 95%CI: 1.14–2.25). Compared to men, women had an estimated 2.8 times higher odds of consuming any medication (OR = 2.79; 95%CI: 2.03–3.84), 3.5 times higher odds of consuming antidepressants (OR = 3.49; 95%CI: 2.25–5.43), 1.9 times higher odds of consuming anxiolytics (OR = 1.89; 95%CI: 1.27–2.81), and 2.4 times higher odds of consuming hypnotics/sedatives (OR = 2.40; 95%CI: 1.64–3.51), adjusting for age, year and education.

**Table 3:** Estimates of the use of psychotropic drugs obtained from multiple Generalised Estimating Equations models

	**OR**	**95% CI**
**Any psychotropic drug**		
**Year**		
2015	1.50	1.13–2.01**
**Gender**		
Women	2.79	2.03–3.84***
**Age**		
≥ 50 at baseline	1.80	1.28–2.54***
**Antidepressants**		
**Year**		
2015	1.37	0.97–1.93
**Gender**		
Women	3.49	2.25–5.43***
**Age**		
≥ 50 at baseline	0.83	0.55–1.26
**Anxiolytics**		
**Year**		
2015	1.22	0.85–1.74
**Gender**		
Women	1.89	1.27–2.81**
**Age**		
≥ 50 at baseline	1.84	1.21–2.79**
**Hypnotics/sedatives**		
**Year**		
2015	1.60	1.14–2.25**
**Gender**		
Women	2.40	1.64–3.51***
**Age**		
≥ 50 at baseline	1.85	1.23–2.79**

Year 2009, gender men and age 18–49 at baseline considered as reference categories across all models

All analysis adjusted for education

** *p* < 0.01

*** *p* < 0.001

Compared to younger individuals, older individuals had an estimated 1.8 times higher odds of consuming any medication (OR = 1.80; 95%CI: 1.28–2.54), anxiolytics (OR = 1.84; 95%CI: 1.21–2.79), and hypnotics/sedatives (OR = 1.85; 95%CI: 1.23–2.79), adjusting for gender, year and education.

The interaction effects of gender and year in the population odds of consuming psychotropic drugs, presented in Table 4, showed that the male population odds of consuming any psychotropic drugs in T1 were estimated to be 1.85 times higher when compared to T0 (OR = 1.85; 95% CI:1.08–3.17) and the odds of the male population consuming hypnotics/sedatives were estimated to be 2.60 times higher in T1 than in T0 (OR = 2.60; 95% CI: 1.36–4.98). The female population odds of consuming any psychotropic drugs in T1 were estimated to be 1.3 times higher than in T0, however without statistical significance (*p* > 0.05). The results also showed that the younger group population odds of consuming any psychotropic drugs (OR = 1.95; 95% CI:1.32–2.90), antidepressants (OR = 1.68; 95% CI:1.05–2.68), and hypnotics/sedatives (OR = 2.16; 95% CI: 1.34–3.47) in T1 were higher when compared to the equivalent consumption in T0. No statistically significant results were found in the older population group.

**Table 4:** Estimates of the interaction effects of gender and year in the population odds of consumption

	**OR**	**95%CI**
**Any psychotropic drug**		
Gender * Year		
Men*2015	1.85	1.08–3.17*
Women*2015	1.34	0.96–1.87
Age * Year		
18–49 at baseline*2015	1.95	1.32–2.90*
≥ 50 at baseline*2015	1.13	0.74–1.71
**Antidepressants**		
Gender * Year		
Men*2015	1.51	0.69–3.31
Women*2015	1.32	0.91–1.93
Age * Year		
18–49 at baseline*2015	1.68	1.05–2.68*
≥ 50 at baseline*2015	0.99	0.61–1.60
**Anxiolytics**		
Gender * Year		
Men*2015	1.49	0.76–2.93
Women*2015	1.07	0.71–1.62
Age * Year		
18–49 at baseline*2015	1.59	0.96–2.65
≥ 50 at baseline*2015	0.92	0.56–1.52
**Hypnotics/sedatives**		
Gender * Year		
Men*2015	2.60	1.36–4.98*
Women*2015	1.26	0.85–1.89
Age * Year		
18–49 at baseline*2015	2.16	1.34–3.47*
≥ 50 at baseline*2015	1.25	0.78–2.01

Year 2009 considered as reference category across all models

All analysis adjusted for education

*confidence interval does not contain value 1

## Discussion

This study provides an assessment of self-reported consumption of psychotropic drugs in 2008/2009 and in 2015/16, which includes the period of economic recession in Portugal, recognizing the importance of gender and age differences. The results show a significant increase in the use of psychotropic drugs during this period, particularly regarding the consumption of hypnotics/sedatives. These findings are in line with other studies about the impact of economic recessions on the consumption of psychotropic drugs [20, 22, 23]. This increase may reflect a deterioration of the mental health of the Portuguese population during the economic recession, as found in epidemiological studies in other countries [13, 14], or higher perceived need of care [39–41].

The results are consistent with other research indicating that women consistently use psychotropic drugs more often than men [26, 27], and these gender differences were found for all the categories of psychotropic drugs [26]. Higher consumption of psychotropic drugs was found in the older age group, also in line with studies that show higher prescription levels with increasing age [27].

However, it is important to highlight that males and younger individuals appear to have been affected disproportionately by the recession in terms of consumption of psychotropic drugs, since the odds of consuming any medication in 2015/16, when compared to 2008/09, were found to be higher in both groups. Higher odds of using hypnotics/sedatives in 2015/16 were found in men, and higher odds of consuming antidepressants, and hypnotics/sedatives were found in younger individuals. These findings are consistent with previous literature suggesting that recessions can be particularly damaging for the mental health of working age men [12, 15, 42]. It has been argued that, during periods of economic recession, the deterioration of mental health outcomes is likely to be associated with individual-level economic shocks (e.g., job and income loss), which men are more likely to experience compared to women [15, 20, 22]. Contributing factors may include shifts in labour markets [15], the disproportionate loss of jobs among men, poor job satisfaction, and an unsatisfactory atmosphere at work [43, 44]. A more pronounced pressure to assume traditional role of breadwinners and for relative socioeconomic success, during a period in life when one may not be fully established in the labour market, offers some additional explanation on why unemployment and uncertainty about the future may have a stronger impact on men’s mental health in recessions [45]. This is particularly important because mental health care utilization patterns differ by gender and seeking help for emotional problems appears to be a more important predictor for the use of psychotropic drugs than a formal DSM diagnosis [39]. Consequently, gender differences in mental health treatment and prescription appropriateness may widen during periods of economic recession, and previous research has shown that men confronted with high job strain used anxiolytics significantly more often than women in similar conditions [26].

Regarding age, the greater increase in prescription drugs utilization between both periods among the younger age group may suggest their increased vulnerability to the economic recession and associated risk factors. Younger workers are exposed to more precarious employment, defined as employment relations characterized by high uncertainty, low income, and reduced social benefits and statutory entitlements [46]. Employment insecurity is associated with poorer mental health [47] and higher probability of psychotropic drugs prescription [46]. Younger individuals were also disproportionately affected by unemployment during the recession in Portugal, with youth unemployment rates of almost 40% in 2014 [48]. Economically inactive groups such as students may also have had a deterioration of their living conditions. Furthermore, young individuals may adopt worse coping strategies to deal with adverse events, with the use of medication being a coping mechanism in times of uncertainty [43] or a compensatory health behaviour in the face of hardship [17].

Several limitations should be acknowledged when interpreting the findings. First, the results were based on self-reported use of psychotropic drugs, which could be subject to recall bias or have been over-reported by one gender or age group compared to the other. Second, the presence of a clinical diagnosis and the appropriateness of prescription were not evaluated, and psychotropic drugs may have been used without a formal DSM diagnosis, for a wide range of emotional problems, or patients with mental disorders may not have been treated with psychotropic drugs [39–41]. Third, the assessment of age through a dichotomous variable was necessary due to the number of individuals in the study but limits the interpretation of results due to the heterogeneity of both groups. Lastly, the analyses did not include terms to account for time trends in drug prescription and/or the net effect of cost-containment pharmaceutical sector policies implemented during recession [11, 28], and it is not possible to state that there was an acceleration in the rate of increase in utilization seen in past decades, nor if this increase was specific to psychotropic drugs.

Despite these limitations, research on the impact of the economic recession on the use of psychotropic drugs is still scarce, and the findings of this study present an innovative contribution to the literature by comparing self-reported consumption of psychotropic drugs, by following the same individuals before and after the economic recession, as well as by assessing differences according to gender and age. Additionally, the time-period of evaluation, which covered the previous 12 months, instead of point or 1- to 2-week prevalence used in most studies, allows to reduce the misclassification of those exposed to treatment, by including both regular users and those discontinuing therapy [26]. This is particularly important for gender comparisons, as women are more likely than men to discontinue treatment in difficult socioeconomic situations [26].

## Conclusions

This study adds to the literature by examining the impact of the 2008/2009 economic recession on the use of psychotropic drugs according to gender and age in Portugal. In line with the existing research, an increase in psychotropic drugs utilization during the period of economic recession was found, with a disproportionate impact on men and younger individuals.

The findings, particularly the increase in the use of hypnotics/sedatives, constitute a public health concern given the already high consumption levels in the country, their limited therapeutic value, and the potential problems of dependence and tolerance. This highlights the importance of defining best prescribing practice recommendations and to invest in psychosocial interventions [49, 50]. Further research is needed to better understand the adequacy of prescribing patterns in Portugal and to design effective public health and labour market policies to mitigate the impact of economic recessions, particularly among vulnerable groups.

## Data Availability

The datasets analysed during the current study are part of the World Mental Health Survey Initiative, are not publicly available and the authors are not authorised to share them.

## Abbreviations

DSM: Diagnostic and Statistical Manual
GDP: Gross Domestic Product
GEE: Generalised Estimating Equations
OR: Odds ratio
WMHS: World Mental Health Survey
